# Media Coverage of Pedophilia: Benefits and Risks from Healthcare Practitioners’ Point of View

**DOI:** 10.3390/ijerph17165739

**Published:** 2020-08-08

**Authors:** Daniela Stelzmann, Sara Jahnke, Laura F. Kuhle

**Affiliations:** 1 Institute of Sexology and Sexual Medicine, Charité – Universitätsmedizin Berlin, 10117 Berlin, Germany; laura.kuhle@charite.de; 2 Institute for Media and Communication Studies, Freie Universität Berlin, 14195 Berlin, Germany; 3 Department of Psychology, Friedrich-Schiller-Universität Jena, 07743 Jena, Germany; sara.jahnke@uni-jena.de

**Keywords:** pedophilia, stigma, media coverage, prevention, media effects, qualitative interviews

## Abstract

The fierce stigma associated with pedophilia may interfere with attempts to prevent sexual offending. Prior research on the effects of media reports about pedophilia mostly focused on their role in perpetuating stigma in the general population. In order to better understand potential benefits and risks of the media coverage on people with pedophilia and specialized prevention and treatment efforts, we conducted semi-structured qualitative interviews with 11 healthcare practitioners of the German Prevention Network “Don’t offend”. Healthcare practitioners described positive (e.g., raising awareness for prevention offers) as well as negative (e.g., perpetuating the existing public stigma) effects of the media coverage and estimated that only about one-third of media coverage portrays pedophilia realistically. To destigmatize pedophilia and benefit the prevention of child sexual abuse, a fact box for journalists was developed based on practitioners’ expert knowledge.

## 1. Introduction

Approximately 1 to 5% of the male population is estimated to have pedophilia [1,2,3], that is, a sexual interest in children [4]. Despite common misperceptions, pedophilia is neither a necessary nor sufficient precondition for sexual offending against children [5]. In fact, an unknown, but most likely substantial number of people with a sexual preference for children never commit sexual offenses [6], and an estimated 40–60 percent of sexual offenses against children are not committed by people with pedophilic interests [5,7,8,9]. Nevertheless, research has shown that people from the general public have extremely negative feelings and attitudes with regard to people with pedophilia, including the ones that do not offend [10]. Accordingly, people with pedophilia have to fear being shamed, threatened, and cut off from sources of social support, if their sexual interests are discovered [6], which may in turn increase the risk of sexual offending and barriers to seeking therapy.

Against this background, having a public that is informed about the difference between pedophilia as a sexual preference versus child sexual abuse as a sexual offense most likely represents an important precondition for the success of preventive measures against child sexual abuse. As one of the main sources of information about pedophilia, the media has a great responsibility to portray pedophilia realistically and to dispel common and harmful myths. Yet, by and large, media reports about pedophilia arguably perpetuate the stereotype that all people with pedophilia are dangerous predators unworthy of our respect or support [11]. In the present article, we will therefore explore the potential risks and benefits of media coverage on pedophilia through the expert opinions of 11 mental healthcare professionals specialized in the treatment of individuals with a pedophilic disorder and the prevention of child sexual offending. Based on these expert opinions, a fact box to evaluate how journalists can improve or impair the mental health of people with pedophilic interests and the prevention of child sexual offending will be proposed.

### 1.1. Risks and Benefits of Media Coverage on Mental Disorders

Media coverage is a crucial source of information that shapes our view of the world [12], especially when first-hand experiences and interpersonal communication are limited, i.e., [13]. Ideally, media reports on mental disorders would provide information based on scientific facts. Such reports have the potential to educate the general public about specific issues like mental disorders and correct prevalent myths about the characteristics and courses of such conditions. This way, media reports can contribute to the destigmatization of mental disorders. For instance, Sampogna and colleagues [14] demonstrated that people who received anti-stigma campaigns via mass media had more knowledge about mental disorders and were more tolerant and supportive of affected persons.

However, media content relies, among other things, on news factors like newness, harm, and oddity, which contribute to the newsworthiness of a story [15,16], and only in part on scientific correctness or evidence-based information [17]. For example, Aragonés and colleagues [18] showed in their study that news reports concerning mental disorders were mostly linked to danger (178 reports out of 362) or violent crime (130 reports out of 362). In an experimental study, Corrigan and colleagues [19] investigated the impact of media coverage on mental disorders. Their results demonstrated that articles which associate mental disorders with negative attributes (e.g., crime) increase stigmatizing attitudes, while decreasing favorable attitudes towards persons with mental disorders.

For this reason, it is not surprising that healthcare practitioners, health advocates, and researchers have repeatedly expressed concern about “both entertainment and news media provid[ing] overwhelmingly dramatic and distorted images of mental illness that emphasize dangerousness, criminality, and unpredictability” (pp. 99, [20], see also [21]). In order to reduce the stigmatization of mental illness, the World Health Organization has identified this topic as a key target for the 2013–2020 action plan [22].

### 1.2. Characteristics of Pedophilia

According to the Diagnostic and Statistical Manual of Mental Disorders (DSM-5) pedophilic interests are not considered pathologic per se [4]. Yet, if a person with pedophilia commits sexual offenses or experiences distress or social difficulties as a result of their sexual interests, the clinical characteristics of a pedophilic disorder are fulfilled [4]. While sexually deviant interests like pedophilia represent a key risk factor for sexual recidivism [23,24], most laypeople and even many mental health practitioners are not aware of the distinction between pedophilia as a sexual interest, pedophilia as a pedophilic disorder, and sexually abusive behavior [25,26]. In fact, the stigmatization of people with pedophilic interests is tremendous [27]. For instance, in a large-scale survey in Germany [9], 14% and 38% of the respondents stated that non-offending people with pedophilic interests should rather be dead or incarcerated, respectively, even if they have never committed a sexual offense. These attitudes towards people with pedophilia are likely to create or exacerbate stress, anxiety, and social isolation [28,29]. Although the stigma attached to pedophilia may be motivated by good intentions (e.g., the desire to protect children), there is also a growing awareness among sexologists and forensic practitioners that public stigma might increase the risk of sexual offenses against children—e.g., by impairing the mental health of persons with pedophilia, or discouraging persons with pedophilia who are at risk for sexual offending from seeking help [5,26,29,30]. Thereby, media coverage which conflates pedophilia and child sexual offending perpetuates the existing public stigma, i.e., [31,32,33].

Therapeutic offers that people with pedophilic interests can turn to are few and far between. The German Prevention Network “Don’t offend” was founded in 2005, and offers free and confidential treatment for people with pedophilia who are seeking therapeutic help (for further information: https://www.dont-offend.org). The therapy aims to prevent both hands-on offenses and offenses related to child sexual abuse images, to support patients to accept and integrate their sexual preference in their self-image, and to reduce psychological distress, i.e., [34,35].

### 1.3. Media Coverage of Pedophilia

Media coverage on pedophilia is often informed by extreme cases of child sexual abuse [32,33]. Catering to viewers’ pre-existing negative ideas about pedophilia, most media coverage cements the stigmatizing assumption that individuals with pedophilia are predatory child sexual offenders and that every child sexual offender has pedophilic interests [11,28,32,36]. The prime example of incorrect reporting in this context is the case of Marc Dutroux, a Belgian who abused and murdered several children, adolescents, and women in the nineties, and whom the media framed as a dangerous pedophile [37], even though expert witnesses came to the conclusion that his acts were motivated by a severe antisocial personality disorder, not pedophilia [38]. On the other hand, there are also evidence-based media reports that correctly inform about the treatment of pedophilic disorder or about child sexual abuse prevention, i.e., [31,32,33,39].

Results of different studies show that educating about a stigmatized minority in the media tends to increase the acceptance of this group in society, i.e., [40], particularly when common myths are addressed and replaced with more accurate information [41]. For instance, the public relations of the German Prevention Network “Don’t offend” pursues the goal of raising problem awareness among people who feel sexually attracted to children and are at risk of committing sexual offenses. Through targeted press and public relations work, people who feel sexual impulses aimed at children are informed about the treatment that is provided by the Prevention Network. Under the campaign motto “To keep fantasies from becoming offenses!”, the program communicates the message that people with pedophilia are not responsible for their sexual attraction but for their behavior, and that there are therapeutic options for those who are afraid of committing sexual offenses. This message is directed both at potential clients with pedophilic interests and the public in order to initiate a more objective and fact-based discussion about pedophilia in society. To achieve this goal, the project is open to cooperation with journalists, i.e., [42].

### 1.4. The Present Research

As of now, little is known about the impact of the media coverage of pedophilia on persons with pedophilia, particularly those in need of treatment. The present study aims to get a deeper understanding of the consequences of the media coverage of pedophilia with respect to stigma, well-being, mental health, treatment, and offense prevention. To this end, we conducted a qualitative interview study with 11 healthcare practitioners at the Berlin site of the Prevention Network “Don´t offend” to investigate the benefits and risks of the media coverage about pedophilia from their point of view. Specifically, we sought answers to the following four research questions (RQ) from healthcare practitioners’ perspectives:

(RQ1) How do therapists perceive the German media coverage about pedophilia, and how well does it correspond to healthcare practitioners’ clinical knowledge?
(RQ2) Which are the most common misconceptions in the media coverage of pedophilia?
(RQ3) What are the benefits and risks of the media coverage of pedophilia in general and for help-seeking pedophiles?
(RQ4) What are newsworthy information on pedophilia from the healthcare practitioners’ point of view?

Based on the results, the present study aims to develop a fact box for journalists in order to ensure a differentiated and destigmatizing media coverage. The complete study was conducted according to the Standards for Reporting Qualitative Research (SRQR, see [43]).

## 2. Methods 

### 2.1. Participants

Previous research [44,45] demonstrated that many healthcare practitioners are not familiar with the treatment of paraphilias, especially pedophilic disorders. This is because paraphilias and their treatment only play a subordinate role in both therapeutic and medical training. Furthermore, not every healthcare practitioner wants to offer treatment for people with a pedophilic disorder [46,47,48]. For this reason, we invited specialized healthcare practitioners (psychiatrists and psychotherapists) from the Berlin site of the Prevention Network “Don’t offend” to participate in the present qualitative study. All healthcare practitioners are experienced therapists who have worked with self-referred, help-seeking people with pedophilic interests for years. At the time of the data collection, the Berlin site of the Prevention Network “Don’t offend” was funded by the German Federal Ministry for Justice and Consumer Protection.

The participants of the study have given full consent to be the case of this study. All information acquired was anonymous, as informants were registered under pseudonyms. This social science study was conducted according to norms of the Code of Ethics of the World Medical Association (Declaration of Helsinki).

A total of 11 healthcare practitioners participated in the study. The participants have worked for the Berlin site of the Prevention Project “Don’t offend” for many years (M = 5.75). Seven of them were psychologists and four medical doctors. Their age ranged from 26 to 55 (M = 36.18), and five of them were female.

### 2.2. Materials

A semi-structured interview including six thematic blocks was developed: 1. healthcare practitioners’ daily work routine, 2. perceived media coverage of pedophilia, 3. the ideal media coverage from participants’ point of view, 4. media coverage and its influence on the barriers to seeking treatment, 5. effects of the media coverage on therapy progress, 6. benefits and risks of the media coverage as well as sociodemographic variables. The interviews were conducted in German. Translated materials are included in Appendix A.

### 2.3. Procedure

All healthcare practitioners were contacted by email. After obtaining informed consent of each participant according to the Declaration of Helsinki, a trained interviewer (first author) conducted semi-structured face-to-face interviews in January 2017. The interview guide is provided in Appendix A. All interviews were recorded with the permission of the participants and subsequently transcribed. According to the Principle of Saturation we closed the data collection after 11 interviews, since the last interviews did not add any new aspects to our research questions. Even though the number of cases seems small, many other studies, i.e., [49,50] have shown that a case number between 10 and 12 persons can be sufficient if the focus is on phenomenological aspects and the group of interviewees is sufficiently homogenous. In such cases, larger samples are not expected to bring any significant added value [51,52].

### 2.4. Data Analysis

Interview data were processed according to Mayring’s [53] qualitative content analysis. In the first step of the analysis, the first author read the entire interviews several times. In the second step, the first author selected statements and their contexts that seemed relevant for answering the research questions. The first author also considered statements and their contexts that did not directly answer the research questions, but imparted other important information. In the third step, the first author paraphrased and generalized all statements and reduced them to categories (for examples, see Table 1). In the fourth step, the first author embedded all identified categories in a broader context of meanings (e.g., quotes). In the final step, the first author discussed the results with the co-authors. In case of discrepancies, corresponding categories and quotations were revisited and their meaning was discussed until a consensus was reached. For all steps of data analysis, the software f4analyse (version 2.5 for iOS; dr. dresing & pehl GmbH, Marburg, Germany) was used.

## 3. Results

In the following, we present the results according to the identified categories and grouped paraphrases.

### 3.1. Perceived Media Coverage and Correspondence with Clinical Knowledge (RQ1)

In order to get a deeper understanding of the media coverage of pedophilia and its consequences, we asked the participants to describe the media coverage from their point of view. From these statements, we were able to identify a total of four central categories, which are described in detail below.

#### 3.1.1. Little Differentiation between Pedophilia and Sexual Offending, but Positive Trend Recognizable 

Most of the 11 participants perceived the German media coverage of pedophilia as undifferentiated. From their point of view, media coverage of pedophilia is usually linked to current cases of child sexual abuse and does not distinguish between pedophilia as sexual interest, a pedophilic sexual preference disorder, and sexually abusive behavior against children (P2: *“So the biggest problem of news reporting is actually still that child abuse (...) is not separated from pedophilia”*). In this context, two participants described the media coverage as unobjective (P3: *"As far as pedophilia is concerned, I miss (...) the necessary objectivity.”*) and two more said that the media coverage is inconsistent (P4: *“So I see two types of reporting”*). Nevertheless, seven participants noted a positive trend towards a more differentiated media coverage over time (P7: *“I think a lot has happened recently. A lot may be exaggerated, but something has changed.”*). One participant thought that this change is largely due to the intense public information campaign for the Prevention Network “Don’t offend”.

#### 3.1.2. Emotionally Charged and Stigmatizing

Even if some of the interviewees described an improvement in media coverage, the majority of therapists were in agreement that the media coverage is, for the most part, emotionally charged and stigmatizing.

P3: *“Emotional and heated.”*

P8: *“Catastrophic (Laughing) would be the honest answer. (...) very undifferentiated, very inflammatory, and very emotional, so little fact-based.”*

About half of the participants (*n* = 5) highlighted that the selective reporting of extreme cases of child sexual abuse (framed as “pedophilia”) turns public opinion against people with pedophilic interests. In this context, participants also invoked recent scandals like the alleged possession of child sexual abuse material by Sebastian Edathy, a prominent former member of parliament [54].

P3: *“It is often very emotional, there is some kind of hook (...). Keyword Edathy (...). It always appears to be very emotionally charged (...).”*

The healthcare practitioners expressed fears that the media contributes to the stigmatization of people with pedophilia with emotionally charged reports that *“lump them [people with pedophilia] together”* (P2, P8, and P10) with people who commit child sexual offenses, and popular frames or metaphors like *“pedophiles are monsters”* (P6).

#### 3.1.3. Definition and Characterization of Pedophilia in the Media versus by Clinicians

Since it could be assumed that the media do not portray pedophilia or people with pedophilic interests accurately, we wanted to know what percentage of media coverage correctly depicts the clinical definition of pedophilia and/or pedophilic sexual preference disorder. For this reason, we asked the interviewees to estimate the percentage of the media coverage that imparts correct information about both. Even though participants found this difficult to answer (*P1: “Very complicated question.”*), all eventually provided an estimate. A slight majority (*n* = 7) estimated that a maximum of one-third of media coverage corresponds with their clinical knowledge. From the clinicians’ point of view, the sensationalist coverage, which does not reflect the clinical picture of pedophilia, prevails.

P1: *“(...) that’s a fraction. One per mill.”*

P2: *“So if you understand pedophilia in the context of international classifications [ICD-10], I guess that’s used correctly in a maximum of 20 percent of cases.”*

P6: *“Not more than 30 percent [evidence-based media coverage about pedophilia].”*

Three participants stated that 50 percent or even more of the media coverage is based on scientific and clinical evidence.

P5: *“I’m not following so intensively, but what I’m seeing... I’d estimate that 70 percent is serious coverage, and the dubious, short-wave coverage is about 30–40 percent.”*

One participant mentioned that people with pedophilic interests who do not sexually offend against children are not interesting enough for reporting.

P2: *“It is very rare that the media presents a pedophile as a pleasant person and it is also rare that there is any reporting at all, unless something has happened. So, if the pedophile has never touched a child, pedophilia is suddenly not an issue anymore. That would be uninteresting, so to speak.”*

#### 3.1.4. The Quality Depends on the Media 

All participants acknowledged differences between at least two types of media coverage: high-quality reports and sensationalist reports. According to the perception of the interviewees, certain journalists deal with the topic and report on it in a nuanced and fact-based manner. In their opinion, these kinds of high-quality reports are well researched by journalists and include essential information about the topic.

P3: *“(...) there are also good articles (...) that really make an effort, which have also spoken with participants [patients from the prevention project that therapists work for], which embed this in figures, and which remain factual.”*

On the other hand, the interviewees also described sensationalist reports. Four participants stated that reports in the so-called yellow press reports were particularly undifferentiated and emotional.

P1: *“[It is] depending on what media you’re looking at. [...] In the yellow press, it is called "The monster from so-and-so” and in the Süddeutsche Zeitung [quality medium in Germany] it does not even make the news.”*

P9: *“But the newspapers or magazines or even television programs, which I would consider to be of higher quality, deal more with the background information than those which are called yellow press.”*

### 3.2. Most Common Misconceptions in the Media Coverage of Pedophilia (RQ2)

Our participants mentioned two typical misconceptions, which are outlined in the following.

#### 3.2.1. No Distinction between Sexual Interest and Sexually Abusive Behavior

Again, all healthcare practitioners stated that the most common misconception in the media coverage of pedophilia is the missing distinction between pedophilia as sexual interest and sexually abusive behavior against children. They emphasized that this is a gross oversimplification with severe and negative consequences for society and people with pedophilia (see also RQ3).

P1: *“Well, the most classic mistake is to equate pedophilia with sexual offence. Which happens again and again...”*

P3: *“The most common mistake is still the equation of pedophilia with child sexual abuse and the assumption that every pedophile man in the first place is driven to commit or wants to harm children. It’s also something you read a lot, and to be honest, I’ve been here for many years [as a therapist] and I haven’t met a patient who wants to harm a child, but they just have that predisposition and have to deal with it.”*

#### 3.2.2. Diagnosing without a Diagnostician

Therapists frequently criticized that journalists often jump to conclusions about the sexual interests of a suspect in a sexual abuse case, labelling that person as a ‘pedophile’ based on criminal actions instead of a careful diagnostic examination. According to the respondents, journalists miss the fact that in many cases, sexual offending is not motivated by corresponding pedophilic interests.

P4: *“For example (...) a person has child sexual abuse images on the computer [...]. It is often concluded that it might have something to do with a sexual interest in children, but in the end, it is not at all clear what is actually the motivation behind this use of child sexual abuse images (...). It is not at all clear if there is a pedophilic motivation behind it or if other things like sadistic fantasies play a role (...). So, these are the most frequent premature speculations [...] in the media.”*

P10: *“So there are perpetrators and understandably, [there is] public indignation about the crimes and it is often then that the pedophilia label comes into play, which is not correct, because perpetrators can be pedophilic but do not have to be and because pedophilic men can become perpetrators but not necessarily.”*

Additionally, some participants noted that journalists might be understandably overwhelmed with the task of writing about a controversial topic like pedophilia without prior clinical or scientific training.

P11: *“That [topic sexuality] sounds as if everybody could write about it, but there’s also a field of ’sexual science’ in the background and to write about it just like that, without the background knowledge, I think it’s also a challenge and that leads, I think, to misunderstandings.”*

### 3.3. Benefits and Risks of the Media Coverage about Pedophilia (RQ3)

The 11 therapists uniformly agreed that from their point of view, the media coverage of pedophilia affects society in general and help-seeking people with pedophilic interests in particular. Furthermore, all of them noted positive and negative consequences, which will be broken down in parts in the following section.

#### 3.3.1. Educating and Destigmatizing 

The interviewees described that media coverage of pedophilia offers two opportunities on a societal level. Firstly, the media confront society with this topic, drawing people’s attention to the fact that pedophilia exists and that it is not *“a marginal phenomenon of society”* (P1). On the other hand, media coverage can educate society about this topic (P9: *"I see opportunities in educating the public.”*). However, from the participants’ point of view, these opportunities only arise when the media coverage of pedophilia includes evidence-based content and handles the topic sensitively and in a destigmatizing way. Also, a few participants described the media dissemination of information about prevention offers like the Prevention Network “Don´t offend” as a powerful tool to generate more beneficial effects through media coverage.

P8: *“So I can see the potential, if you [the media] approached it in a more fact-based way and less emotional.”*

P3: *“Destigmatization in the sense of “constant dripping is wearing down the stone”. One can only emphasize: nobody chooses it [pedophilia], but it is there, what are we going to do with it, etc.”*

#### 3.3.2. Perpetuating the Existing Social Stigma in Society

Participants uniformly stated that the existing stigma by no differentiation between pedophilic interests and sexually abusive behavior against children, and emotionalization of the topic is perpetuated by media coverage.

P5: *“I think the media can contribute very much to (...) destigmatization just as they can contribute very much to stigmatization.”*

P4: *“Risks arise above all from journalists not knowing the tools of their trade well enough or knowing them but consciously using them in a somewhat different way and not communicating certain nuances and attempting to get a sensationalist headline.”*

#### 3.3.3. Raising Awareness and Increasing the Self-Reference to Therapy 

Respondents stated that media coverage of pedophilia raises awareness for those who have these sexual interests. From their point of view, media coverage helps some pedophilic individuals become aware of problematic aspects of their sexuality.

P4: *“Simply raising awareness that there might be a problem can be achieved by people becoming aware of the media coverage (...) and then thinking ‘there might be a problem with me’.”*

Furthermore, ten therapists observed that media coverage prompts potential patients to seek contact with the Prevention Network “Don´t offend”. According to the respondents, this effect was most pronounced when independent journalists cooperated with the treatment project, or when media campaigns were launched by the project itself. Hence, it was noted that the Prevention Network “Don´t offend” depended on the media reporting on it:

P3: *“Yes, the contacts correlate with ads in major newspapers. Whenever something is in the media, more people contact us. So, we are dependent on being featured in the media.”*

P7: *“At the same time, when good articles are published or good interviews are done, more patients contact us. That means you can reach a lot of people.”*

The positive correlation between media coverage of pedophilia (especially when the Prevention Network “Don´t offend” was involved in reporting) and number of patients seeking contact led many to conclude that media coverage can also help overcome the barrier to seeking therapy.

P4: *“And the second thing is, if you find out that there’s a professional offer available, it could definitely lower your inhibitions about confiding in a healthcare professional.”*

#### 3.3.4. Increasing Therapy Barriers and Decreasing Self-Esteem

Most of the therapists stated that media reporting on pedophilia, particularly in the context of low-quality tabloid coverage, can negatively affect the motivation of people with pedophilia to seek therapy.

P5: *“There were people who introduced themselves to us, who have known about the project for a long time and have the feeling that this might be something for me and who, due to the stigmatization they experience in public, and adding the current media coverage, has prevented them from contacting us. With others it was exactly the other way around.”*

The therapists repeatedly mentioned the stigmatization of people with pedophilic interests, which is exacerbated by various media. According to the interviewees, this stigmatizing media coverage stops people with pedophilic interests from seeking help and increases their level of distress.

P6: *“Stigmatization then already keeps patients from contacting us at all. (...) It [media coverage] also helps because people no longer want to be stigmatized and then need therapy in our project. But it also keeps them away because even with us or anyone else, they don’t dare to speak about what is really going on inside.”*

Moreover, participants believed that the sensationalist and stigmatizing media reports influence the self-perception of those affected and increase their fear of disclosing.

P4: *“Yes, of course, very sensationalistic reporting (...) can of course lead one to thinking ‘Oh God, I better not out myself.’”*

P2: *“Conversely, of course, people will feel stigmatized, they will have difficulty turning to us, because the image they have of themselves is ‘the monster’, ‘the felon’. Even if that doesn’t correspond to reality. So, it has an impact, of course. It mainly has an influence on the self-perception of the people concerned.”*

Participants hypothesized that it is through its negative influence on self-perception and the fear of coming out, that the stigma conveyed by the media leads to social isolation among people with pedophilia. Furthermore, social isolation, in turn, represents a risk factor for committing child sexual abuse (hands-on as well as hands-off offenses), as one participant mentioned.

P2: *“Risk is stigmatization, which is not good for anyone, because stigmatization is followed of course by loneliness. And loneliness is a risk factor for, again in the broadest sense, child sexual abuse or the use of child sexual abuse images.”*

Although the media coverage may have a negative influence on the acceptance of prevention offers, several interviewees pointed out that they mainly deal with people with pedophilic interests who have already overcome this barrier.

P1: *“I don’t know the people who don’t come to us. I know that it is a problem for many people who are with us that pedophilia is so stigmatized. And therefore, inhibitions are growing about opening it up to someone else. But those who come to us have overcome this barrier.”*

#### 3.3.5. Consequences for People with Pedophilia during the Course of Their Treatment

Since the patients of the Prevention Network “Don’t offend” have already overcome the barrier to self-referring to therapy, we wanted to know about further effects that the media coverage might have on people with pedophilic interests during the course of treatment.

##### Patients with Pedophilia Suffer from the Stigmatizing Media Coverage 

The therapists agreed that media coverage affects their patients in other respects as well. Mainly, participants described that stigmatizing through media coverage increases their patients’ distress, self-stigma, and fear of coming out.

P8: *“So this goes as far as suicidal thoughts”*

P2: *“Well, I assume that a large part of the media coverage contributes to stigmatization and from that we know that our patients feel much more stigmatized (...) this naturally has an influence on their self-esteem (...) and that they think they are much bigger monsters than society [does].”*

##### Dealing with the Stigmatizing Media Coverage Often Becomes Part of Therapy 

Therapists reported that the media coverage of pedophilia typically becomes a recurring theme within the weekly group therapy sessions. They pointed out that this is particularly likely when media reports about a prominent case of child sexual abuse or child sexual abuse image offending (e.g., Sebastian Edathy) or a change in the law occurs. In this context, patients and therapists would typically discuss the public image of ‘the pedophile’ and whether and how much patients identify with it. If the media coverage on this topic is intense, they noted that patients became more afraid that others may discover their sexual interests and felt more discriminated. The participating healthcare professionals reported that they attempted to encourage patients to critically scrutinize stigmatizing articles and to remind themselves how media processing works (e.g., focusing only on people who offend against children).

P3: *“[Patients] who tell us ‘We feel like we are denounced as monsters, just because of these sexual interests.’”*

P4: *“[This] I have to answer with ’no’, but also because we do not provide any conceptual recommendations. But it [media coverage] is addressed and from it how they sort this information for themselves into their world view.”*

P11: *“Nothing specific, except that we’re going to discuss how media coverage works.”*

### 3.4. Newsworthy Information on Pedophilia from the Therapists’ Point of View (RQ4)

The participants stated that excellent reporting on pedophilia is characterized by a nuanced, objective, nonjudgmental, and destigmatizing portrayal. Furthermore, respondents highlighted four facts that journalists should address in their reporting, which are described in the following.

#### 3.4.1. Difference between Sexual Preference and Sexual Behavior against Children 

Everyone agreed that the media should inform that having sexual interests in children and sexual offenses against children are not the same thing.

P2: *“Well, whenever the word pedophilia comes up, it should be written in big letters (...) as an introduction: “Pedophilia is not child sexual abuse and child sexual abuse is not pedophilia. Or just a definition box. ‘What is pedophilia?’”*

P1: *“The difference between sexual preference and sexual behavior should be addressed again and again if possible.”*

#### 3.4.2. Condemn Offending, Do not Condemn Fantasies 

Furthermore, some respondents pointed out that reporting the differentiation between sexual preference and sexual behavior against children also means to distinguish between the levels of sexual fantasy and sexual behavior. In contrast to sexual fantasies with children, they emphasized, adults’ sexual behavior with children should always be condemned, regardless of the sexual preference of the offender.

P4: *“In sexuality, there is a big difference between what happens in fantasies or on the imaginary level and what we actually act out on the behavioral level.”*

P8: *“Fantasy is not behavior.”*

#### 3.4.3. Not a Marginal Phenomenon in Society

Although people with pedophilia are often considered to be very rare, some of the respondents (*n* = 3) considered it important to point out that having sexual fantasies involving children is not a rare phenomenon in society.

P1: *“And the fact that it is not a marginal phenomenon of society.”*

#### 3.4.4. No One Chooses His or Her Sexual Preference

Participants argued that since people with pedophilic interests are rejected by many people in society and are often accused of having deliberately chosen this sexual preference, media reporting should highlight that nobody chooses these sexual interests and that no one deserves to be judged or blamed because of it.

P7: *“That it starts with puberty, which means not only among people with 50+ years, that it can actually happen to anyone and that no one deliberately chooses it.”*

## 4. Discussion

This study used qualitative methodology to gain insight into the characteristics and effects of the current media coverage of pedophilia in Germany. To this end, we interviewed 11 healthcare professionals of the Berlin site of the Prevention Network “Don’t offend” with regards to their perception of the media coverage of pedophilia (RQ1), common misconceptions (RQ2), benefits and risks of the media coverage of pedophilia (RQ3), and facts that journalists should be aware of when writing about pedophilia (RQ4).

Our participants described the media coverage of pedophilia as undifferentiated, emotionally charged, and stigmatizing. Yet, our results also indicate considerable differences in media depictions of people with pedophilia. This is in line with previous research, i.e., [31], which observed that media reports about pedophilia do not portray this phenomenon realistically. In particular, the study participants criticized the common equation of pedophilia with child sexual abuse by labeling a person as a ‘pedophile’ based on criminal offenses rather than a thorough clinical assessment of the defendant’s sexual interests. This is a common concern in the literature on stigmatization and pedophilia in many Western countries, i.e., [11,32,33,36]. Nevertheless, participants also perceived a positive trend towards more fact-based media reports in Germany throughout the last few years. 

In line with the literature on the stigma of mental illness or sexual offending, i.e., [14,19,21,40], our results suggest that media coverage can both educate society and perpetuate stigmatizing beliefs. Strikingly, our results indicate that even negative reporting may have benefits, at least compared to no reporting, because it may raise awareness of their sexuality among people with pedophilia. Nevertheless, from the point of view of the therapists in this sample, accurate reports on pedophilia are seen as most beneficial. This corresponds to several studies demonstrating a positive impact of an objective and benevolent media coverage on public attitudes towards people with mental disorders or people who have sexually offended, i.e., [14,40]. 

In line with the news value theory [15,16], our findings suggest that people with pedophilia are primarily the subject of discussion in the context of current high-profile cases of child sexual abuse. Although media reporting in Germany now seems to be more balanced and also reports on existing prevention offers, the media still tends to conflate pedophilia and child sexual abuse, while non-offending people with pedophilia are not newsworthy for journalists. The clinicians in the current sample were uniformly critical of this type of reporting, as they noted that it put people with pedophilia at risk of emotional distress through the internalization of stigmatization and fears of being rejected. Put another way, this means that the one-sided and undifferentiated media coverage is likely to contribute to the development of a pedophilic disorder by triggering distress symptoms like shame or anxiety in relation to pedophilic interests.

Accordingly, a survey by Cohen and colleagues [55] revealed that more than one-third of their community sample of participants with pedophilia reported chronic suicidal ideation. Against that background, it seems indispensable that non-offending pedophiles are given a higher news value to counteract one-sided media coverage, which may add to psychological distress. Nevertheless, nuanced and fact-based reporting can only emerge if journalists receive expert help and guidance when writing about topics that are fraught with emotion and stigma. The successful adoption of expert guidelines for journalists has been demonstrated in other areas (e.g., guidelines for reporting on suicide that were developed by the World Health Organization [56]). It is therefore important to develop guidelines for media coverage of pedophilia and to distribute them to journalists’ associations. 

Furthermore, our results suggest various negative influences of the media coverage especially on those people with pedophilic interests who are seeking therapeutic help. This corresponds with other quantitative and qualitative studies that indicated a negative effect of public stigma on people with pedophilic interests [26,29,30], while adding important observations on how the media reporting in particular may add to this stigma. With regards to the influence of media stigmatization on the barrier to self-referring to treatment, our study provides further valuable insights. While a previous quantitative study did not find a significant correlation between stigma-related stress and therapy motivation among a non-clinical sample of men with pedophilia [28], therapists in the present study assumed that stigmatizing media reports decrease the willingness to seek therapy among people with pedophilic disorder. If their assumptions are correct, destigmatizing media content represents a viable route to decrease therapy barriers from the participants’ point of view. Yet, little is known about the effect of different types of media reporting on willingness to pursue treatment among people with pedophilia. Hence, to gain a better understanding of the effects of media reporting, further research may experimentally investigate the effects of different types of media coverage on increasing therapy motivation among people with pedophilic disorder. In this context it seems essential to investigate the framing of the articles. As discussed above, media outlets typically report about treatment offers for people with pedophilia in the context of child sexual abuse prevention, which implies that people with pedophilia need therapy primarily to prevent them from sexually offending against children. This kind of prevention framing may not reflect the actual needs of many non-offending people with pedophilia. According to recent studies [29], many people with pedophilic disorder are primarily looking for help to learn how to deal with psychosocial problems and psychological distress and not because they see themselves at risk of committing child sexual abuse offenses. An additional or alternative framing, which focuses on the psychological needs of the affected persons and not on child sexual abuse prevention, could inform non-offending people with pedophilia about therapeutic approaches that better suit their mental health goals.

Compared to other countries, German laws protect therapist/client confidentiality, including information about paraphilic fantasies and potential past sexual offenses. This confidentiality creates a safe environment that encourages help-seeking people with pedophilia to confide in a therapist. Consequently, primary prevention programs against child sexual abuse like the “Don’t offend” project or other programs specialized in the treatment of people with pedophilic disorders could establish themselves relatively easily in Germany compared to countries with, e.g., mandatory reporting laws. We therefore suggest that future research should examine the media coverage on pedophilia in other countries and media systems, where we might expect an even greater lack of evidence-based reporting on pedophilia and less availability and awareness of treatment opportunities for pedophilic disorder. This makes it all the more important to investigate how the media in other countries report on pedophilia and which social rooms people with pedophilic interests create for themselves (i.e., web forums) when no or only limited therapeutic options are available.

To improve the quality of the media coverage of pedophilia, participants highlighted four key facts that should be mentioned in every article on the topic that is targeting a general audience. Again, the equation of pedophilia with child sexual abuse has been singled out as the most pertinent point by the study participants, since it perpetuates the stigma attached to pedophilia and may cut people with pedophilia off from sources of social and emotional support. Therefore, therapists recommend that media reports need to explain the difference between pedophilia as sexual interest and sexual offending against children. As suggested by Harper and Harris [57], using the term ‘people with sexual interests in children‘ instead of ‘pedophile‘ to avoid the association with people who sexually offend can be a first step towards decreasing the conflation between the two concepts, see also [58]. Moreover, in the context of reporting on child sexual abuse cases, journalists should only use the term pedophilia/pedophilic disorder if there is sufficient evidence (typically based on psychiatric or sexological expert witness assessment) that the defendant actually has a sexual interest in children. Unless such information is available, journalists should refrain from using potentially false diagnostic labels, and instead use more accurate terminology like “the accused/defendant”. Journalists may also use this opportunity to remind their readers that sexual offending behavior is not a reliable indicator of pedophilia, as a considerable proportion of sexual offenses against children are committed by people without a sexual preference for children. 

Additionally, participants stated that it is essential to communicate that sexual fantasies and sexual behavior are different concepts and not synonymous. This is in line with research that demonstrates that people who have sexual fantasies about harmful or illegal acts (as in the case of fantasies about sexual aggression) overwhelmingly do not act on these fantasies, i.e., [59]. 

Furthermore, pedophilia is not as rare as many may presume it is and should therefore not be reported as a marginal phenomenon [1,2,3].

Previous research found that people with stigmatized identities (e.g., mental disorders) are often blamed for having this stigma by others, which may lead to both social rejection and social withdrawal [60,61,62]. This makes it all the more important to communicate that people with pedophilic interests do not choose their sexual interests, as no one else does.

## 5. Limitations

Although the results are intriguing, they are based on a small and highly selective sample of clinicians from the Berlin site of the Prevention Network “Don’t offend”. As noted by the study participants themselves, they only had clinical experiences with a subsample of people with pedophilia, namely those who self-referred to the project and who experienced psychological distress and/or saw themselves at risk for sexual offenses. Hence, they cannot provide insights regarding people with pedophilia who are not in need of therapy or seek other therapeutic options. Also, they cannot inform our perspective regarding people with pedophilia who sexually offend against children or are at risk of doing so but are not problem-aware and not motivated to seek treatment. In particular, participants are likely to have limited contact with people with pedophilia who do not experience distress due to their sexual interests or are not concerned about acting on their sexual impulses (e.g., because they have sufficient behavioral control). Furthermore, therapists would not have contact with people with pedophilia who are too afraid to contact the Prevention Network “Don’t offend” due to their fear of stigmatization and also the fear that they might be recognized and identified or other reasons (e.g., limited mobility, lack of time) that might keep potential clients from contacting the “Don’t offend” Network. This might have led to therapists over- or underestimating the potential negative effect of stigmatizing media reports on willingness to pursue therapy. Future studies that analyze the impact of media coverage on people with pedophilic interests in non-clinical settings may remedy some of these shortcomings, as these might be able to reach participants who might benefit from therapy but have not sought therapeutic help so far.

Qualitative designs can help to further our understanding of a given subject and/or generate new hypotheses. In our case, the descriptive approach enabled us to gain valuable insights about the complex relationship between media stigmatization, symptoms of distress, and therapy acceptance among people with pedophilia, which should be further explored in larger and more controlled quantitative studies. Yet, reducing the interviews to a selection of paraphrased codes and thematic frames can lead to a loss of information or idiosyncratic meaning [63]. Furthermore, we cannot determine the strength or significance of an effect, nor establish causality based on our results. Hence, we recommend the use of quantitative measures and experimental settings to test the causal effects of stigmatizing reports with more methodological rigor, i.e., [21].

## 6. Conclusions

The present study aimed to yield a deeper understanding of the consequences of media coverage of pedophilia for the mental health of people with pedophilia and their help-seeking behavior. The results suggest that media coverage of pedophilia has a range of potential risks and benefits. Most importantly, the emotionalized, stigmatizing, and uniformly negative media coverage of pedophilia, which is common in the yellow press (i.e., no differentiation between pedophilia as sexual preference, pedophilic disorder as a mental disorder, and sexual offenses against children) appears to be most harmful.

**Figure 1:**
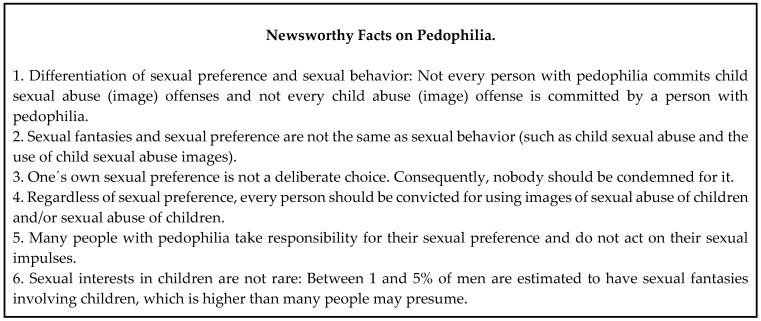
Newsworthy Facts on Pedophilia.

Ischebeck and Stelzmann [64] argue that many misconceptions of pedophilia are due to a lack of knowledge among journalists. Some journalists in their survey stated that they would like to have more expert support regarding this topic. In line with this, our findings indicate that cooperations between journalists and clinical experts can transform the way that journalists write about mental disorders. For this reason, both mental health practitioners who are sexologically qualified and treat people with a pedophilic disorder and journalists should actively cooperate to increase the quality of media reporting about pedophilia, pedophilic disorder, and child sexual abuse. Furthermore, it seems appropriate, as with other mental illnesses, to formulate guidelines for journalists. For example, the World Health Organization published detailed recommendations on how to report ethically about suicide (e.g., “Provide information about where to seek help”) [56]. As a first step to support an ethically sensitive reporting about pedophilia, we propose a fact box (Figure 1), which includes newsworthy facts on pedophilia from the experts’ point of view.

## Figures and Tables

**Table 1:** Example data analysis.

Quote	Paraphrase	Generalizability	Reduction/Category
“Well, the classic mistake is to equate pedophilia and sexual offense. Which happens again and again. Otherwise typical mistakes are made: diagnosing without a diagnostician (...).”	a classic mistake is to equate pedophilia and sexual offense; typical mistakes are made: diagnosing without a diagnostician	No differentiation between offense and preference; diagnosing without a diagnostician	No distinction between sexual interest and sexually abusive behavior Diagnosing without a diagnostician

## Appendix A

**Questionnaire**

Introduction:

Hello Mr./Ms. XY, first, thank you for taking the time to answer a few of my questions. As you have already heard from Mr. XXX, my colleagues and I—from the Institute for Media and Communication Studies—are currently researching the media coverage (in general: print, TV, online) about pedophiles/pedophilia. Within this context, I would like to talk to you, among other things, about the way you experience the media coverage about pedophiles/pedophilia, if it has effects on therapy, etc.

For simplicity, I would like to record the interview. The collected data are only used for scientific purposes and will be analyzed anonymously, so that no conclusion to your person will be possible. Do you agree with this?

1. Before we start talking about the media coverage, I would firstly like to know how you started working for the project “Don´t offend“. Perhaps you could say a few words about that.

Ask, if not mentioned:

1.1 What is your exact job description?

1.2 How does your daily occupational routine look like?

1.3 How many patients are you currently working with?

2. When thinking about the media coverage about pedophiles/pedophilia in general, how would you describe it?

Ask, if not mentioned:

2.1 Could you give me a short example that—from your perspective—represents the typical media coverage?

2.2 Are there differences within the media coverage? (Bild vs FAZ, exemplification vs. objective)

2.3 How strong is the distortion in your view?

*Alternatively: Does the media coverage correspond with your professional view?*

2.4 What are the typical/most common mistakes that occur in media coverage?

*Alternatively: Are there any differences, when you compare a typical media report on pedophilia with your professional view?*

2.5 If you had to estimate now: What percentage of media coverage reflects reality? *Alternatively:* clinical picture

2.6 What problems do you see in the way the media report?

2.7 Studies have shown that the subject of pedophilia is strongly stigmatized. What role does the media play in this? (reinforcement, education, etc.).

2.8 How intensively do you deal with media coverage yourself? How does this apply to your daily life: How often do you read about it?

3. What do you think the media coverage should look like?

Ask, if not mentioned:

3.1 Is there any information that should be communicated in each article, report, etc.?

3.2 And if so, what information about pedophilia do you think should always be reported on?

4. Do you think that the media coverage—in general—has an influence on the acceptance of therapy opportunities?

4.1 What influence does it have?

4.2 Why does it have that influence?

4.3 The inhibition to take up a therapy opportunity is usually quite high. Do you think the media coverage has influence on that?

4.5 If so, in which form?

5. Apart from a possible inhibition of therapy acceptance, do you have the feeling that the media coverage has a concrete influence on your patients?

5.1 If so, what does that look like?

5.2 Are/were there moments when you talk/talked very specifically with patients about the media coverage? What was it about?

5.3 Do you give your patients recommendations on how to deal with that media coverage?

6. Finally, I would be interested to know what opportunities and risks you see in the media coverage of pedophilia.
